# Hepatitis E Infections, Victoria, Australia

**DOI:** 10.3201/eid1103.040706

**Published:** 2005-03

**Authors:** Benjamin C. Cowie, Jim Adamopoulos, Karen Carter, Heath Kelly

**Affiliations:** * Royal Melbourne Hospital, Parkville, Victoria, Australia;; † Department of Human Services, Melbourne, Victoria, Australia;; ‡ Victorian Infectious Diseases Reference Laboratory, North Melbourne, Victoria, Australia

**Keywords:** dispatch, hepatitis E virus, communicable diseases, serologic tests, epidemiology, tropical medicine, public health, disease outbreaks, travel, Victoria, Australia

## Abstract

In the first half of 2004, acute hepatitis E virus infections diagnosed in Victoria, Australia, increased 7-fold. Of the interviewed patients with highly reactive serologic results, 90% reported recent clinically compatible illness and overseas travel. The increase is compared with a background of exposure in countries in which hepatitis E is endemic.

Hepatitis E virus (HEV) is a major cause of enterically transmitted hepatitis worldwide. It is an important pathogen in Asia, the Middle East, and parts of Africa and Central America (1,2). Epidemic HEV is manifested in waterborne outbreaks, often involving thousands of people, which predominantly occur in areas where environmental sanitation facilities are inadequate. However, endemic (sporadic) HEV accounts for the majority of infections (2). In India, HEV is responsible for 50% to 70% of all cases of sporadic viral hepatitis (1).

HEV seroprevalence in disease-nonendemic areas such as Australia is low, ≈1% to 2% (3–5), in contrast to disease-endemic areas where seroprevalence increases as residents increase in age from 10% to 40% in adults (1,3,5,6). Some of this seropositivity is explained by subclinical infection and by persistence of immunoglobulin (Ig) G against HEV, which has been detected <14 years after infection (7).

The incubation period for HEV is 2 to 9 weeks, and the spectrum of disease ranges from subclinical infection to fulminant hepatitis. Clinical features can include fever, chills, jaundice, dark urine, anorexia, nausea, vomiting, abdominal pain, headache, myalgia, and arthralgia (1–3).

In general, HEV infection is more likely to be subclinical in children (1). Although usually a self-limiting disease with death rate <1%, a high incidence of fulminant hepatitis is seen in pregnant women, in whom the death rate can exceed 20% in the third trimester (2,3). HEV does not lead to persistent infection or chronic hepatitis (1,2). No vaccine is currently available.

In disease-nonendemic countries such as Australia, almost all patients with HEV infection report recent travel to areas where the disease was endemic (1,2,5). New evidence, however, suggests that HEV is more prevalent in industrialized countries than previously thought (8) and that zoonotic transmission may be implicated (1,2,5).

As in many jurisdictions, hepatitis E must be reported as an infectious disease throughout Australia. In Victoria, the Department of Human Services must be notified by both the treating doctor and the testing laboratory. In the first 6 months of 2004, an increase in positive hepatitis E serologic results was observed at the Victorian Infectious Diseases Reference Laboratory, a state reference and public health laboratory. Investigations to determine whether these serologic results represented true acute hepatitis E infections were conducted.

## The Study

To fulfill the Victorian hepatitis E case definition for surveillance purposes, one must demonstrate seroconversion, a 4-fold rise in paired serum specimens or detect highly reactive IgG in a single specimen in the presence of a clinically compatible illness (9). Two commercial tests have been used for HEV serologic testing at the Victorian Infectious Diseases Reference Laboratory, the Abbott HEV enzyme immunoassay (EIA) (Abbott GmbH Diagnostika, Wiesbaden-Denkenheim, Germany) before March 2004 and the Genelabs HEV enzyme-linked immunosorbent assay (ELISA) (Genelabs Diagnostics Pte Ltd., Singapore) after March 2004. These tests detect anti-HEV IgG in the patient's serum by using recombinant antigens from the structural region of the HEV genome (3,4). Internal Victorian Infectious Diseases Reference Laboratory validation confirmed concordance of results using these tests, and both produced highly reactive results in this case series. Specimens are referred to the Victorian Infectious Diseases Reference Laboratory from private pathology laboratories, hospitals, and general practitioners throughout Victoria and also from other states and countries.

The EIA result is expressed as the ratio of the absorbance of the patient sample to the assay cut-off absorbance (s/co). A sample with a ratio of >1.0 is considered positive. A high s/co ratio indicates highly reactive patient serum, which suggests recent infection (2,6,10). Anti-HEV Ig G titers peak from 2 to 4 weeks after disease onset (11,12) and diminish relatively rapidly thereafter (2,8,10–12). For the purposes of this study, we have defined highly reactive results as those with an s/co ratio of >5.0, which has been associated with recent infection (12) and the presence of anti-HEV Ig M (13). Testing for antihepatitis E Ig M was not performed.

**Figure 1:**
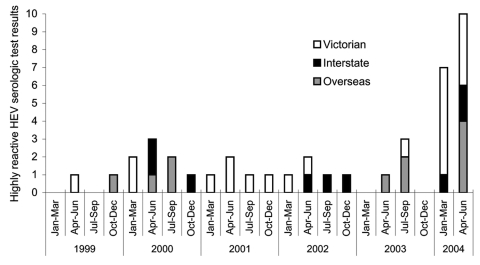
Highly reactive hepatitis E virus (HEV) enzyme-linked immunosorbent assay results at the Victorian Infectious Diseases Reference Laboratory per quarter, January 1, 1999, to June 30, 2004.

For the first 2 quarters of 2004, 7 and 10 highly reactive (s/co>5.0) hepatitis E EIA results were found, respectively. Both figures were the highest to date, and well above 1.2, the quarterly mean number of highly reactive results for the previous 5 years (p < 0.0001, χ^2^ test) (Figure 1). Ten highly reactive specimens were from Victorian patients, compared to an expected number of 1.1 for the 6-month period (p < 0.0001). Total positive results (all s/co > 1.0) for the first 2 quarters of 2004 were also significantly increased at 7 (p = 0.0103) and 12 (p < 0.0001) compared to 2.75, the quarterly mean for the previous 5 years.

All highly reactive HEV EIA results detected at the Victorian Infectious Diseases Reference Laboratory are reported to the Department of Human Services. Victorian case-patients (those whose specimens are referred for testing by a Victorian doctor) are investigated by means of a standard questionnaire administered through telephone contact with both the patient and his or her doctor to determine clinical details, travel history, and other information. For patients outside Victoria, the appropriate authorities are notified wherever possible.

The 17 highly reactive samples tested at the Victorian Infectious Diseases Reference Laboratory during the first 2 quarters of 2004 accounted for all hepatitis E notifications in the state during this period. However, only 2 of these patients were also notified by the treating doctor, as required by legislation.

Nine of the 10 Victorian patients reported having been in a disease-endemic area within the incubation period and also having experienced an illness clinically compatible with HEV infection (Table). The remaining patient reported no compatible illness and, therefore, did not meet the Victorian case definition for surveillance. He last travelled in a disease-endemic area (Pakistan) in 2001.

**Table:** Clinical and epidemiologic information on Victorian hepatitis E case-patients January 1 to June 30, 2004

Age	Sex	Symptoms	Onset date*	Date of test	Countries visited during incubation period	Duration of visit	Comments
51	F	Jaundice, dark urine	01/04/04	01/11/04	India	NA	Indian resident recently arrived in Australia.
26	M	Fever, jaundice, dark urine, nausea, vomiting	02/13/04	03/01/04	India, Sri Lanka, Thailand, Singapore	20 weeks, 2 weeks	Thailand and Singapore were overnight stopovers only.
18	M	Jaundice	02/18/04	03/02/04	India	4 weeks	Did not make contact with DHS.† Information provided by doctor. Patient HBcAb+.‡
28	F	Fever, jaundice, dark urine	02/21/04	02/24/04	India	7 weeks	
20	F	Jaundice, dark urine, abdominal pain, nausea	03/13/04	03/23/03	India, Thailand, China	11 weeks, 2 days, 5 weeks	Worked in a Children's Hospital in India.
22	M	Jaundice, dark urine, abdominal pain, vomiting, diarrhea	03/25/04	03/25/04	India	NA	Indian resident recently arrived in Australia.
41	M	Dark urine, fever, abdominal pain, nausea, vomiting, diarrhea, headache, myalgia	05/06/04	05/06/04	Thailand	3 weeks	Reported swimming in a lake that locals warned was polluted.
60	F	Fever, dark urine, abdominal pain, nausea, diarrhea, headache, myalgia	05/13/04	05/26/04	Vietnam	13 weeks	Worked in an orphanage in Vietnam.
45	M	Jaundice, nausea	05/19/04	05/19/04	India	12 weeks	
28	M	None	NA	06/24/04	No recent travel history	NA	Last traveled to disease-endemic area, Pakistan, in 2001. Tested for hepatitis E (amongst other investigations) by local doctor because of mild liver function test abnormalities.

*Date of first appearance of jaundice or dark urine. †Department of Human Services. ‡Hepatitis B core antibody positive.

Except for 1 patient whose serum tested positive for antibodies to hepatitis B core antigen, no positive serologic results for other hepatitis viruses (including hepatitis A) were reported in the patients from Victoria. Four of the 7 specimens from non-Victorian residents had been collected from patients from India and sent by a private laboratory to the Victorian Infectious Diseases Reference Laboratory, and 3 specimens were from patients from other states.

## Conclusions

We found a 7-fold increase in the number of serum samples that were highly reactive for anti-HEV Ig G tested at the Victorian Infectious Diseases Reference Laboratory in the first half of 2004, from a mean for the last 5 years of 2.4, to 17 (p < 0.0001). Ten of these specimens were from patients in Victoria, a notable increase from the mean number for the previous 5 years of 1.1 for the 6-month period (p < 0.0001). As is characteristic in a disease-nonendemic region, 9 of the 10 highly reactive Victoria serum samples tested in this period were from patients who had recently traveled in disease-endemic countries, namely, India, Sri Lanka, Thailand, and Vietnam.

Why HEV infections in Victoria have recently increased cannot be established with certainty. Possible explanations include an increase in the number of tests performed, an increase in HEV activity in the countries visited by the travelers, an increase in the number of travelers to or from disease-endemic areas, and changes in behavior among the travelers. The last 2 possibilities are beyond the scope of this article but merit further consideration. The first 2 are explored further.

**Figure 2:**
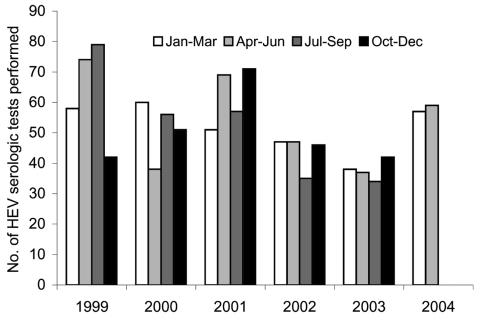
Total number of hepatitis E virus (HEV) enzyme-linked immunosorbent assays performed at the Victorian Infectious Diseases Reference Laboratory per quarter, January 1, 1999, to June 30, 2004.

The mean number of hepatitis E serologic tests performed at the Victorian Infectious Diseases Reference Laboratory per quarter over the previous 5 years was 51.6 (Figure 2). In the first 2 quarters of 2004, respectively, 57 and 59 tests were performed, which is not significantly different from what was seen in previous quarters (p = 0.452 and 0.303).

Overseas hepatitis E activity does appear to have increased during this period. ProMED-mail, the global electronic reporting program for emerging diseases hosted by the International Society for Infectious Diseases, reported >800 cases of hepatitis E infection in eastern Calcutta, India, on April 23, 2004 (14). Contaminated water pipelines were implicated. Other areas with hepatitis E outbreaks reported by ProMED-mail in the first half of 2004 included Bangui, Central African Republic (6/19/2004), Punjab, Pakistan (6/6/2004), Sadr, Iraq (6/2/2004), and Gujarat, India (5/5/2004).

The first confirmed outbreak of hepatitis E with ≈29,000 cases of hepatitis occurred in Delhi in 1955–1956 when raw sewage contaminated drinking water during heavy flooding (2,11,14). Other epidemics include Kashmir in 1978, with an estimated 52,000 cases of hepatitis and 1,560 deaths (8), and the largest epidemic on record in northwest China in 1986–1988 with >100,000 cases (15).

Increased diagnoses of hepatitis E in Victorian travelers may have provided "early warning" of an evolving outbreak in an HEV-endemic area, particularly if a similar increase is reported in other non–HEV-endemic areas. Residents of resource-rich, non–disease-endemic countries such as Australia likely have greater access to hepatitis E testing than those living in resource-poor, HEV-endemic areas where the greatest incidence of this disease occurs.

Persons traveling to developing countries must be advised of preventive measures they should take against hepatitis E and other enterically transmitted diseases. Hepatitis E infection should be considered in any febrile person who has recently traveled in a disease-endemic area, particularly if jaundice or abnormal liver function tests are found, and especially in pregnant women due to the risk of fulminant hepatitis. Cases should be reported to public health authorities according to local legislation.
